# Supplementary material for: A chimeric Mla-Pqi lipid transport system is required for Brucella abortus survival in macrophages
Source: EMBO J. 2025 Aug 13;44(18):5066–85. doi: 10.1038/s44318-025-00511-3 (PMC12436622; doi:10.1038/s44318-025-00511-3)
Supplement: Supplementary file 1 — Appendix [file 44318_2025_511_MOESM1_ESM.pdf]

Appendix for

A chimeric Mla-Pqi lipid transport system is required for *Brucella abortus* survival in macrophages

Table of contents

Item	Page
Appendix Table S1. List of strains used in this study	2
Appendix Table S2. List of plasmids used in this study	2
Appendix Table S3. List of primers used in this study	3
Appendix Table S4. List of ORFs used in this study	4
Appendix Table S5. DOC-sensitive mutants according to Tn-seq	5
Appendix Figure S1. Growth of *mpc* mutants inside the host cell	6
Appendix Figure S2. Decreased of cardiolipin (CL) in OMVs in the *mpc* mutants	6
Appendix Figure S3. Lipid composition of whole cells compared to outer membrane vesicles	7
Appendix Figure S4. Multiple sequence alignment of Mpc proteins	8
Appendix Figure S5 Multiple sequence alignment with Mce complexes from *Mycobacterium tuberculosis* and *Arabidopsis thaliana*	9
Appendix Figure S6. The 5 model interactions predicted for the Mpc complex	11
Appendix Figure S7. Structural comparison of MpcA/PqiC and MpcD/MlaD	13

Appendix Table S1. List of strains used in this study

Name	Description and relevant genotype	Reference
	Brucella abortus 544	
Wild type (WT)	*B. Abortus* 544, Nal^{R}	J-M. Verger, INRA, Tours
Δ*mpcE*	*B. abortus* 544 Δ*mpcE*	This study
Δ*mpc*F	*B. abortus* 544 Δ*mpcF*	This study
Δ*mpc*D	*B. abortus* 544 Δ*mpcD*	This study
Δ*mpc*A	*B. abortus* 544 Δ*mpcA*	This study
Δ*mpc operon*	*B. abortus* 544 Δ*mpc operon*	This study
Δ*mpcE* pMR10 *mpcE*	*B. abortus* 544 Δ*mpcE* pMR10 *mpcE*	This study
Δ*mpcF* pMR10 *mpcF*	*B. abortus* 544 Δ*mpcF* pMR10 *mpcF*	This study
Δ*mpcD* pBBR2 *mpcD*	*B. abortus* 544 Δ*mpcD* pBBR2 *mpcD*	This study
Δ*mpcA* pBBR2 *mpcA*	*B. abortus* 544 Δ*mpcA* pBBR2 *mpcA*	This study
mpcA-StrepTag	*B.abortus* 544 *mpcA*-StrepTag	This study
Δ*cls*	*B. abortus* 544 Δ*cls*	This study
Δ*mpc operon* Δ*cls*	*B. abortus* 544 Δ*mpc operon* Δ*cls*	This study
Δ*olsA*	*B. abortus* 544 Δ*olsA*	This study
Δ*mpc operon* Δ*olsA*	*B. abortus* 544 Δ*mpc operon* Δ*olsA*	This study
Δ*olsB*	*B. abortus* 544 Δ*olsB*	This study
Δ*mpc operon* Δ*olsB*	*B. abortus* 544 Δ*mpc operon* Δ*olsB*	This study
Δ*asmA*	*B. abortus* 544 Δ*asmA*	This study
Δ*mpc operon* Δ*asmA*	*B. abortus* 544 Δ*mpc operon* Δ*asmA*	This study
	Escherichia coli	
DH10B	F– *mcrA* Δ(*mrr-hsdRMS-mcrBC*) ϕ80*lacZ*ΔM15 Δ*lacX74 recA1 endA1 araD139* Δ (*ara-leu*)7697 *galU galK* λ– *rpsL*(Str^{R}) *nupG*	Invitrogen
S17-1	M294::RP4-2 (Tc::Mu)(Km::Tn7)	Simon *et al*., 1983
MFD*pir*	MG1655 RP4-2-Tc::[ΔMu1::aac(3)IV-ΔaphA-Δnic35-ΔMu2::zeo] ΔdapA::(erm-pir) ΔrecA	Ferrières *et al*., 2010
MFD*pir* pXMCS-2 mini-Tn*5* Kan^{r}	MFD*pir* pXMCS-2 mini-Tn*5* Kan^{r}	This study

Appendix Table S2. List of plasmids used in this study

Name	Reference
pNPTS138	M. R. K. Alley, Imperial College of Science,London, UK
pBBR2 MCS	Deghelt *et al*., 2014, Unamur, BE
pMR10	C.D. Mohr and R.C. Roberts, Stanford University, US
pXMCS-2 mini-Tn*5* Kan^{r}	Sternon *et al*., 2018, Unamur, BE
pNPTs 138 Δ*mpc*E	This study
pNPTs 138 Δ*mpc*F	This study
pNPTs 138 Δ*mpc*D	This study
pNPTs 138 Δ*mpc*A	This study
pNPTs 138 Δ*mpc* operon	This study
pMR10 *mpc*E	This study
pMR10 *mpc*F	This study
pBBR2 MCS *mpc*D	This study
pBBR2 MCS *mpc*A	This study
pNPTs 138 mpcA-Strep Tag	This study
pNPTs 138 Δ*cls*	This study
pJQ200 Δ*olsA*	Palacios-Chaves *et al*., 2011, Universidad de Navarra, Spain
pJQ200 Δ*olsB*	Palacios-Chaves *et al*., 2011, Universidad de Navarra, Spain
pNPTs 138 Δ*asmA*	This study
pBBR2 MCS *asmA*	G. Potemberg, PhD thesis, Unamur, BE

Appendix Table S3. List of primers used in this study

construction	Name	Reference
Δ*mpcE*	P53_F_AM_a1055	ACGGCATGGATACGTTCAATGTCG
	P54_R_AM_a1055	AGGGGATTCCGGCCTACCTCATATACGAGTCGTTGA
	P55_F_AV_a1055	GAGGTAGGCCGGAATCCCCTCAATTGGGCGC
	P56_R_AV_a1055	GAAAACAGCGCGCCATGCTG
Δ*mpcF*	P57_F_AM_a1056	GTCCGATCTCTCCGCACTTGTG
	P58_R_AM_a1056	TTTCCATATCTCTTTAACCGAGCTTGCTGCCAG
	P59_F_AV_a1056	CGGTTAAAGAGATATGGAAACAAAAGCCAATTACGTAC
	P60_R_AV_a1056	CACGAAACCTTCAAGCTGGC
Δ*mpcD*	P61_F_AM_a1057	GATGGCCATCGATATGCGCATG
	P62_R_AM_a1057	TTTGGAAATATCTTATCCAGTCTGCAAGCGGT
	P63_F_AV_a1057	TGGATAAGATATTTCCAAAACCTGTTCCAGTGACATG
	P64_R_AV_a1057	AGCCTTCACATAGGCCGCAT
Δ*mpcA*		GCTGAGCCAGCTTGAAGGTTTC
	P65_F_AM_a1058	CGGAACCGCTGTCACTGGAACAGGTTTTGGAAATCAA
	P66_R_AM_a1058	TG
	P67_F_AV_a1058	TTCCAGTGACAGCGGTTCCGGTTAAAACGGA
	P68_R_AV_a1058	CGGTTGAGCTTTGGGACCGTTAT
Δ*mpc* operon	P69_R_AM_operon	CGGAACCGCTGGCCTACCTCATATACGAGTCGTTGA
	P70_F_AV_operon	GAGGTAGGCCAGCGGTTCCGGTTAAAACGGA
pMR10 *mpcE*	P97_F_comp-mpcE_KpnI	GCGggtaccCAATGTATTGTGCATAGAAGTGCCCTTC
	P98_R_comp-mpcE_SacI	GCGgagctcCTAGAAATTAATTGCCGCATAAAACATT
pMR10 *mpcF*	P99_F_comp-mpcF_KpnI	GCGggtaccGAGACACAGTTGCACATAAAATTGACG
	P100_R_comp-mpcF_SacI	GCGgagctcTTATCCAGTCTGCAAGCGGTTG
pBBR2 *mpcD*	P102_F_comp-mlaD	TGCGCGACGTTACGGTTAAGCGTGCGCGTCAGATT
	P103_R_comp-mlaD_SacI	GCGgagctcTCAATGACGCTTCCTCCCATC
pBBR2 *mpcA*	P104_F_comp-mlaA	TGCGCGACGTTACGGTTGCCGGTGAGTGTGCCTCAAT
	P105_R_comp-mlaA_SacI	GCGgagctcCTAAAGGGTTGAAATCGTCCAGCTTACG
mpcA-Strep Tag	P303_mpcA_F	GCGctgcagATGAAATCACGGCGCATGTTCCGTC
	P304_mpcA_R	agccaccgccAAGGGTTGAAATCGTCCAGCTTACGATC
	P305_mpcAST_F	TTCAACCCTTggcggtggctcgggcgg
	P331_mpcAST-R	CGGAACCGCTCTActtttcgaattggggatgagaccac
	P332_mpcAST-AV-F	cgaaaagTAGAGCGGTTCCGGTTAAAACGGA
	P333_mpcAST-AV-R	CATCTGCGGAAAAGCCGCG
Δ*cls*	P382_AM_F_CLS	CGGAACCGATCAATGCGGC
	P383_AM_R_CLS	GCGCGATCGACCAGCAAACTTTAAGCAAAAAAATGGC
	P384_AV_F_CLS	AGTTTGCTGGTCGATCGCGCTATATTGTAGCGC
	P385_AV_R_CLS	GCTGGCACGGCAATGTGC
	P386_F_CLS_check2	TGGCAATGATAAGCCCCAGCA
	P387_R_CLS_check2	GCCGATGTCATTGCGCTCCA
Δ*olsA*	P420_OlsA_F_check	AGGACAGCCACGCCATCC
	P421_OlsA_R_check	TCCTGCCCCTCGGCCT
Δ*olsB*	P422_OlsB_F_check	TGCAGTATTCAATCTTGCAGATGCATTC
	P423_OlsB_R_check	GTGGTGAACCGTGCACGC
Δ*asmA*	P418_F_check_Asma	CACTCTTGTGGATTGAATTTGACGTCTG
	P419_R_check_Asma	AGGTCCGCCTTGGTGTGAC

Appendix Table S4. List of ORFs used in this study

Brucella abortus 544	*Brucella abortus* 2308		UniProt	
ORF ID	ORF ID	Gene name	Accession number	Predicted function
BAB_v1_a0027	BAB1_0022		Q2YPN7	conserved protein of unknown function
BAB_v1_a0090	BAB1_0084	*ybhL*	Q2YNU5	Bax inhibitor-1/YccA family protein
BAB_v1_a0115	BAB1_0108	*ndvB*	Q2YNV7	Cyclic beta-(1,2)-glucan synthase NdvB
**BAB_v1_a0155**	**BAB1_0147**	***olsB***	**Q2YNY9**	**L-ornithine N(alpha)-acyltransferase**
BAB_v1_a0287	BAB1_0279	*btpA*	Q2YPC4	Probable 2' cyclic ADP-D-ribose synthase BtpA
BAB_v1_a0316	BAB1_0304	*cenK*	Q2YPD1	Histidine Kinase
BAB_v1_a0333	BAB1_0322	*bepD*	Q2YPE7	Efflux pump periplasmic linker BepD
BAB_v1_a0334	BAB1_0323	*bepE*	Q2YPE6	multidrug efflux pump RND permease AcrB
BAB_v1_a0362	BAB1_0351	*wadB*	Q2YPI9	Glycosyl transferase, family 25
BAB_v1_a0516	BAB1_0507	*pdeA*	Q2YML1	Sensory box/GGDEF domain/EAL domain protein
BAB_v1_a0742	BAB1_0722	*omp25*	Q2YN33	25 kDa outer-membrane immunogenic protein
BAB_v1_a0979	BAB1_0963	*bepC*	Q2YNS2	Outer membrane efflux protein BepC
BAB_v1_a1032	BAB1_1017	*ndvA*	Q2YQ73	Beta-(1-->2)glucan export ATP-binding/permease protein NdvA
**BAB_v1_a1055**	**BAB1_1038**	***mpcE***	**Q2YQ52**	**STAS domain-containing protein**
**BAB_v1_a1056**	**BAB1_1039**	***mpcF***	**Q2YQ51**	**ABC transporter ATP-binding protein**
**BAB_v1_a1057**	**BAB1_1040**	***mpcD***	**Q2YQ50**	**TOLUENE TOLERANCE PROTEIN TTG2C**
**BAB_v1_a1058**	**BAB1_1041**	***mpcA***	**Q2YQ49**	**ABC transporter**
BAB_v1_a1076	BAB1_1063	*nrdJ*	Q2YQ30	Vitamin B12-dependent ribonucleotide reductase
BAB_v1_a1102	BAB1_1092		Q2YQ08	conserved exported protein of unknown function
BAB_v1_a1196	BAB1_1177		Q2YRQ1	conserved protein of unknown function
BAB_v1_a1420	BAB1_1404	*pdxJ*	Q2YQM7	Pyridoxine 5'-phosphate synthase
BAB_v1_a1552	BAB1_1538	*petR*	Q2YRL4	Protein PetR
**BAB_v1_a1641**	**BAB1_1623**	***asmA***	**Q2YRB2**	**AsmA family protein**
BAB_v1_a1886	BAB1_1866		Q2YLK9	Peptidase, M23/M37 family
BAB_v1_a1972	BAB1_1956	*abcP*	Q2YLW4	Amino acid ABC transporter, permease protein
BAB_v1_a2013	BAB1_1996	*ftsX*	Q2YR73	membrane protein of unknown function
BAB_v1_a2015	BAB1_1997	*ftsE*	Q2YR72	Cell division ATP-binding protein FtsE
BAB_v1_a2021	BAB1_2006	*cenR*	Q2YR63	DNA-binding response regulator
BAB_v1_a2038	BAB1_2025	*dnaJ*	Q2YR47	heat shock protein DnaJ
**BAB_v1_a2166**	**BAB1_2153**	***olsA***	**Q2YQS9**	**Lyso-ornithine lipid O-acyltransferase**
BAB_v1_b0021	BAB2_0021		Q2YL88	putative anti-sigma-F factor NrsF
BAB_v1_b0182	BAB2_0185	*yqjF*	Q2YIJ7	Inner membrane protein YqjF
BAB_v1_b0718	BAB2_0727	*cydB*	Q2YKD5	cytochrome bd-I ubiquinol oxidase subunit II
BAB_v1_b0719	BAB2_0728	*cydA*	Q2YKD4	cytochrome bd-I ubiquinol oxidase subunit I

Appendix Table S5. DOC-sensitive mutants according to Tn-seq. For each mutated gene, a Transposon insertion frequency (TnIF) is computed. Transposon insertion sites were identified through Illumina sequencing. In the control condition, there was an average of one unique insertion site every 2.62 bp, and in the envelope stress condition, every 2.48 bp, saturating the *B. abortus* genome in both conditions. Following reads mapping, we computed an ES.Reads value for each open reading frame (ORF), corresponding to the insertion number per bp for 80% of each ORF by excluding the first and last 10% of the predicted coding sequence. To enable a quantitative analysis of each ORF under different conditions, we calculated a transposon insertion frequency (TnIF) parameter. This frequency corresponds to the logarithm in base 10 of the mapped read numbers for the central 80% of each ORF (log_{10} (ES.Reads+1)). To identify genes required for growth in the presence of DOC (0.015%), we compared the TnIF of the stress condition to the control condition to obtain a ΔTnIF for each ORF ($\Delta TnIF = TnIF_{DOC} - TnIF_{Ctrl}$). The standard deviation (SD) of ΔTnIF values for the all the ORFs corresponds to 0.323. We considered ORFs with less than one SD (*; - 0.323) as less required, two SD (**; -0.647) as required, three SD (***; -0.97) as highly required and four SD (****; -1.293) as required for growth on DOC.

			TnIF(normalised) : log10 (ES.reads+1)		ΔTnIF		
ORF ID	ORF ID	Gene name	Ctrl	DOC	DOC		Predicted function
**BAB_v1_a1057**	**BAB1_1040**	***mpcD***	**4.520**	**1.079**	**-3.441**	****	**TOLUENE TOLERANCE PROTEIN TTG2C**
BAB_v1_a2021	BAB1_2006	*cenR*	4.339	1.380	-2.958	****	DNA-binding response regulator
**BAB_v1_a1058**	**BAB1_1041**	***mpcA***	**4.454**	**1.826**	**-2.628**	****	**ABC transporter**
**BAB_v1_a1055**	**BAB1_1038**	***mpcE***	**4.542**	**2.182**	**-2.360**	****	**STAS domain-containing protein**
BAB_v1_a0979	BAB1_0963	*bepC*	4.449	2.338	-2.110	****	Outer membrane efflux protein BepC
**BAB_v1_a1056**	**BAB1_1039**	***mpcF***	**4.348**	**2.486**	**-1.862**	****	**ABC transporter ATP-binding protein**
BAB_v1_a1886	BAB1_1866	*rgsM*	4.350	2.718	-1.633	****	Periplasmic peptidase
BAB_v1_a0316	BAB1_0304	*cenK*	4.624	3.264	-1.360	****	Sensory Transduction Protein Kinase
BAB_v1_a0334	BAB1_0323	*bepE*	5.161	4.162	-0.999	***	multidrug efflux pump RND permease AcrB
BAB_v1_a1102	BAB1_1092		4.211	3.282	-0.929	**	conserved exported protein of unknown function
BAB_v1_a0333	BAB1_0322	*bepD*	4.920	4.068	-0.852	**	Efflux pump periplasmic linker BepD
BAB_v1_a1641	BAB1_1623	*asmA*	5.049	4.201	-0.848	**	AsmA family protein
BAB_v1_a0516	BAB1_0507	*pdeA*	4.671	3.851	-0.820	**	Sensory box/GGDEF domain/EAL domain protein
BAB_v1_a0362	BAB1_0351	*wadB*	4.208	3.491	-0.717	**	Glycosyl transferase, family 25
BAB_v1_a1552	BAB1_1538	*tcbR*	4.556	3.865	-0.692	**	Two component response regulator
BAB_v1_b0021	BAB2_0021		3.956	3.318	-0.638	*	putative anti-sigma-F factor NrsF
BAB_v1_a1196	BAB1_1177		2.983	2.346	-0.636	*	conserved protein of unknown function
BAB_v1_b0718	BAB2_0727	*cydB*	4.428	3.880	-0.547	*	cytochrome bd-I ubiquinol oxidase subunit II
BAB_v1_a0090	BAB1_0084	*ybhL*	4.299	3.768	-0.532	*	Bax1-I family protein YbhL
BAB_v1_a1032	BAB1_1017	*ndvA*	4.409	3.914	-0.495	*	Beta-(1-->2)glucan export ATP-binding/permease protein NdvA
BAB_v1_a2013	BAB1_1996	*ftsX*	4.042	3.582	-0.460	*	membrane protein of unknown function
BAB_v1_b0719	BAB2_0728	*cydA*	4.514	4.068	-0.446	*	cytochrome bd-I ubiquinol oxidase subunit I
BAB_v1_b0182	BAB2_0185	*yqjF*	4.432	3.994	-0.439	*	Inner membrane protein YqjF
BAB_v1_a2015	BAB1_1997	*ftsE*	4.049	3.625	-0.424	*	Cell division ATP-binding protein FtsE
BAB_v1_a1420	BAB1_1404	*pdxJ*	3.911	3.494	-0.417	*	Pyridoxine 5'-phosphate synthase
BAB_v1_a0287	BAB1_0279	*btpA*	5.748	5.347	-0.402	*	NAD(+) hydrolase BtpA
BAB_v1_a0027	BAB1_0022		4.011	3.615	-0.396	*	conserved protein of unknown function
BAB_v1_a0742	BAB1_0722	*Omp25*	4.193	3.810	-0.383	*	25 kDa outer-membrane immunogenic protein
BAB_v1_a1076	BAB1_1063	*nrdJ*	4.936	4.586	-0.350	*	Vitamin B12-dependent ribonucleotide reductase
BAB_v1_a2038	BAB1_2025	*dnaJ*	4.078	3.729	-0.349	*	heat shock protein DnaJ
BAB_v1_a0115	BAB1_0108	*cgs*	4.967	4.621	-0.346	*	Cyclic beta-(1,2)-glucan synthase NdvB
BAB_v1_a1972	BAB1_1956	*abcP*	5.120	4.795	-0.326	*	Amino acid ABC transporter, permease protein

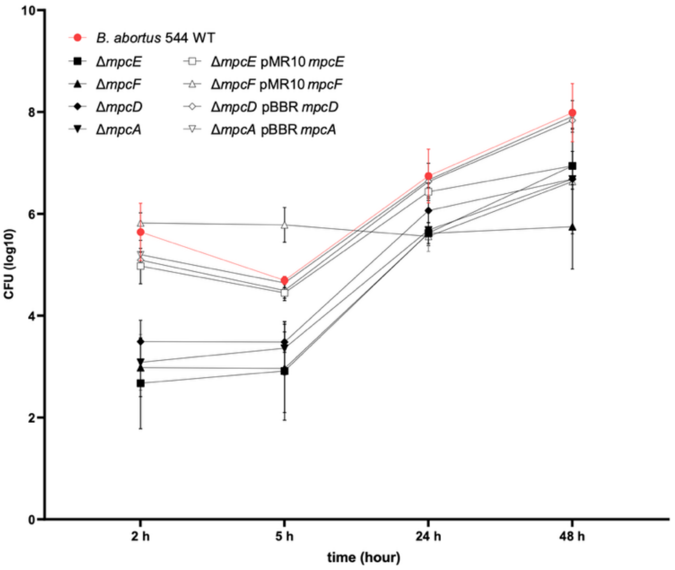

Appendix Figure S1. Growth of *mpc* mutants inside the host cell. Intracellular replication of WT, *mpc* mutants and the complemented strains were assessed by counting colony-forming units (CFU) at 2, 5, 24, and 48 hours post-infection of J774.A1 macrophages. The data represents the mean ± SD and were compiled from three independent replicates.

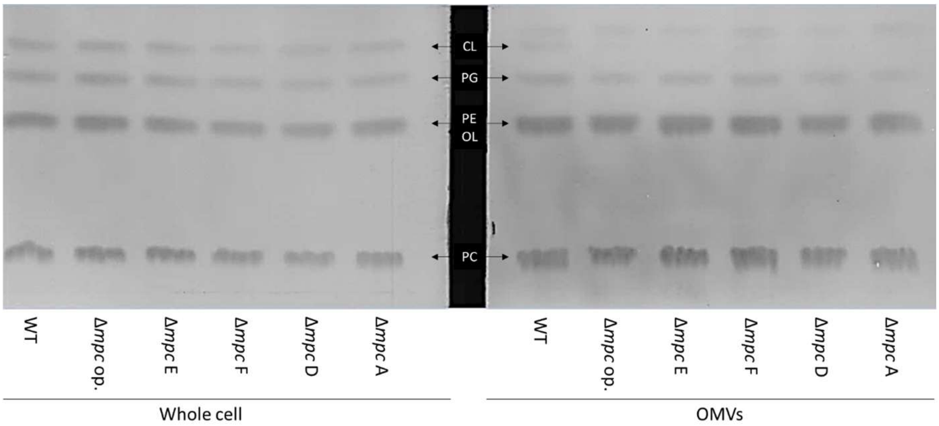

Appendix Figure S2. Decreased of cardiolipin (CL) in OMVs in the *mpc* mutants. Thin layer chromatography (TLC) was performed on lipid extracts obtained from both whole cells and outer membrane vesicles (OMVs). The lipids were separated by phosphatidylcholine (PC), phosphatidylethanolamine and ornithine lipids (PE/OL), phosphatidylglycerol (PG) and Cardiolipin (CL) from bottom to the top. In the OMVs samples a band appeared above the CL, but the nature of this lipid is unknown and could not be revealed by staining with iodine vapor.

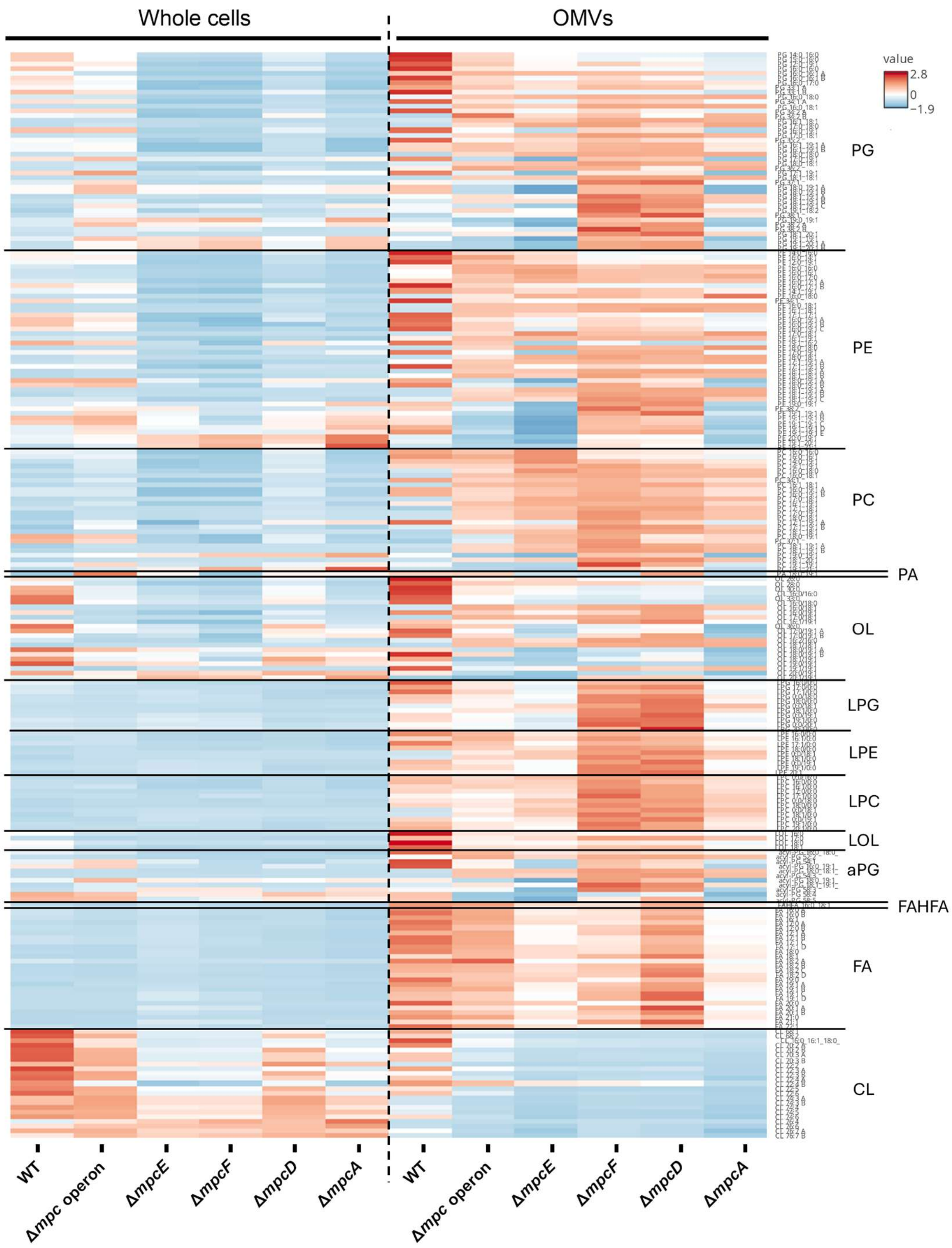

Appendix Figure S3. Lipid composition of whole cells compared to outer membrane vesicles. Heat map of abundance of phospholipids identified by mass spectrometry-based lipidomic analysis. PG: phosphatidylglycerol, PE: phosphatidylethanolamine, PC: phosphatidylcholine, PA: Phosphatidate, OL: ornithine lipid, LPG: lyso-phosphatidylglycerol, LPE: lyso-phosphatidylethanolamine, LPC: lyso-phosphatidylcholine, LOL: lyso-ornithine lipid, aPG: aminoacyl- phosphatidylglycerol, FAHFA: fatty acid esters of hydroxy fatty acid, FA: fatty acid, CL: cardiolipin.

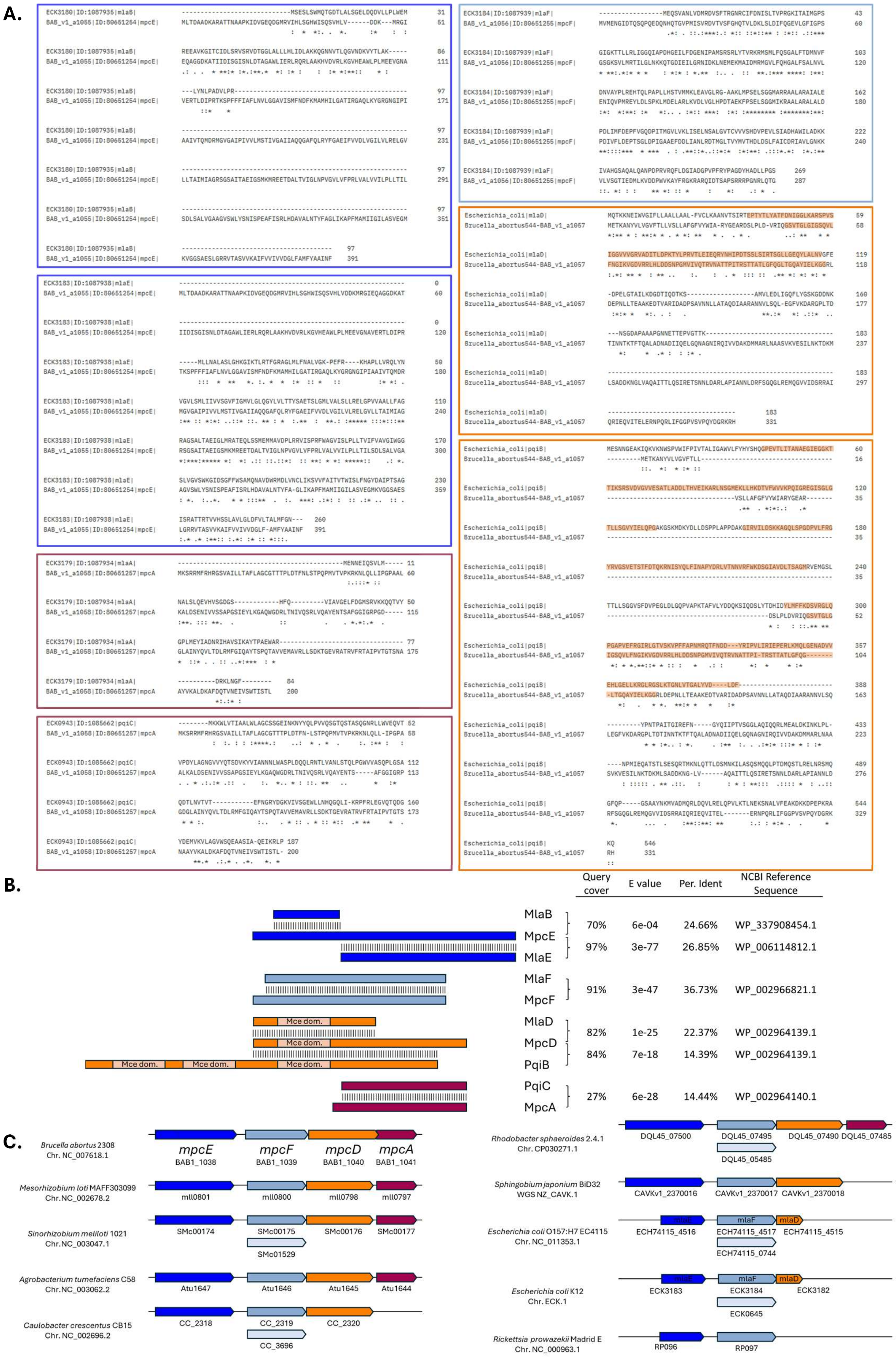

Appendix Figure S4. **Multiple sequence alignment of Mpc proteins. A.** Sequence alignments of proteins of Mla and Pqi complex to Mpc complex. The sequence highlighted in orange correspond to the MCE domain. **B.** Homology of proteins of Mla and Pqi complex using BlastP on the *Brucella* genome

with the query cover, the e-value and the percentage of identity **C.** Synteny of operon encoding for the Mpc system of *Brucella abortus* 2308
https://mage.genoscope.cns.fr/microscope/mage/viewer.php?label=3940687&KeepTailleZone=True

A.

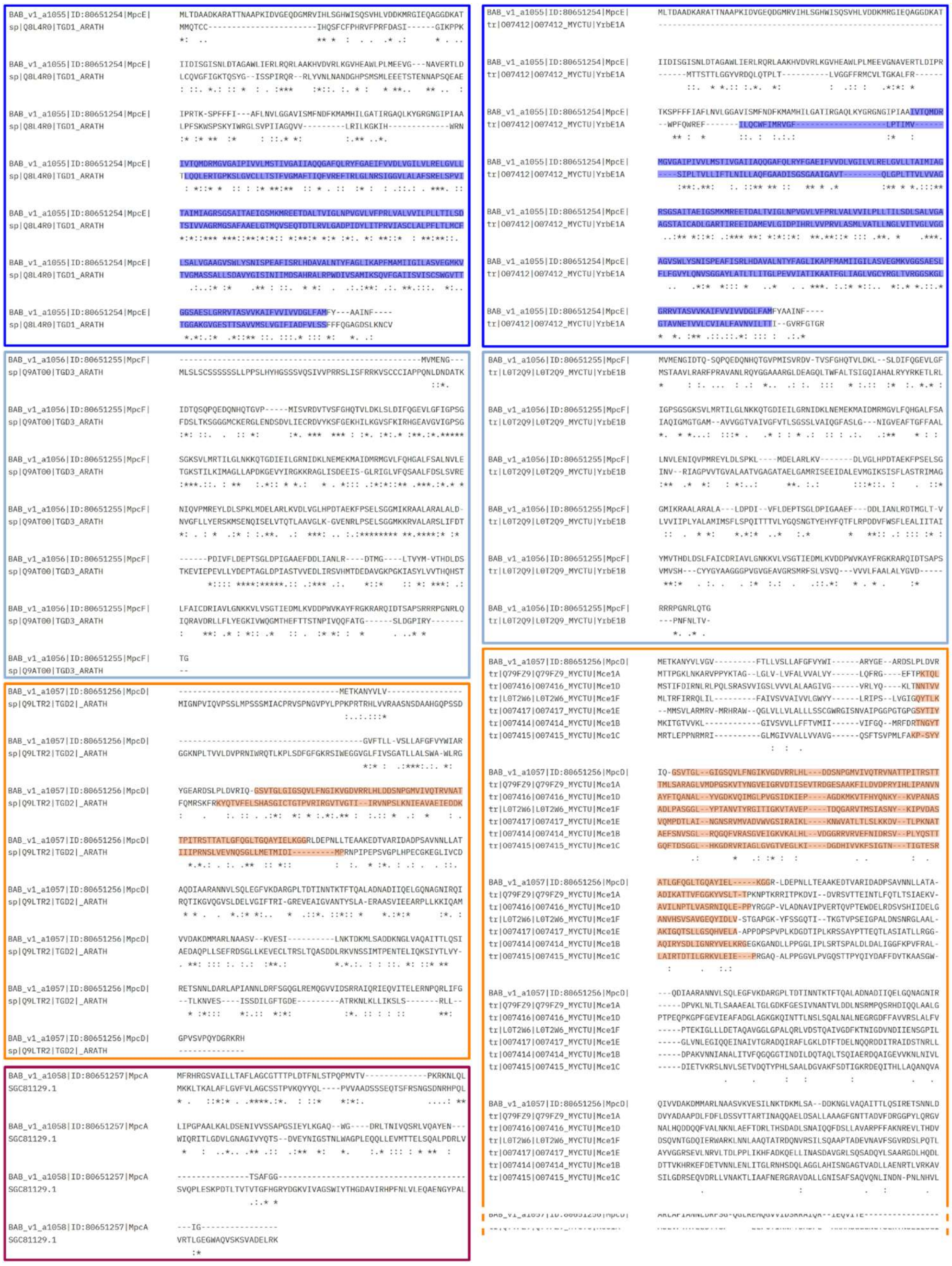

B.

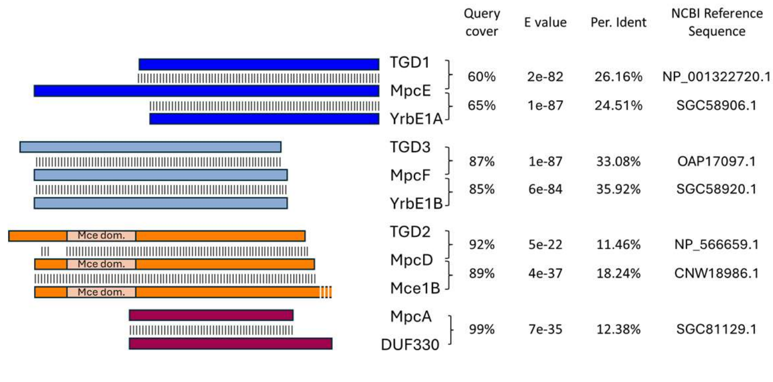

Appendix Figure S5 [previous page]. **Multiple sequence alignment with Mce complexes from *Mycobacterium tuberculosis* and *Arabidopsis thaliana*. (A.)** Protein sequence alignments of Mce1 and TGD complexes to Mpc complex, respectively from *Mycobacterium tuberculosis* and *A. thaliana*. The sequences highlighted in orange correspond to the MCE domain and in dark blue correspond to the permease MlaE domain (Pfam PF02405). **(B.)** Homology of proteins of the Mpc system using using BlastP on the *Mycobacterium tuberculosis* (taxid:1773) or *A. thaliana* (taxid:3702) genomes with the query cover, the e-value and the percentage of identity. No homologue for the MpcA was identified in *A. thaliana* genome.

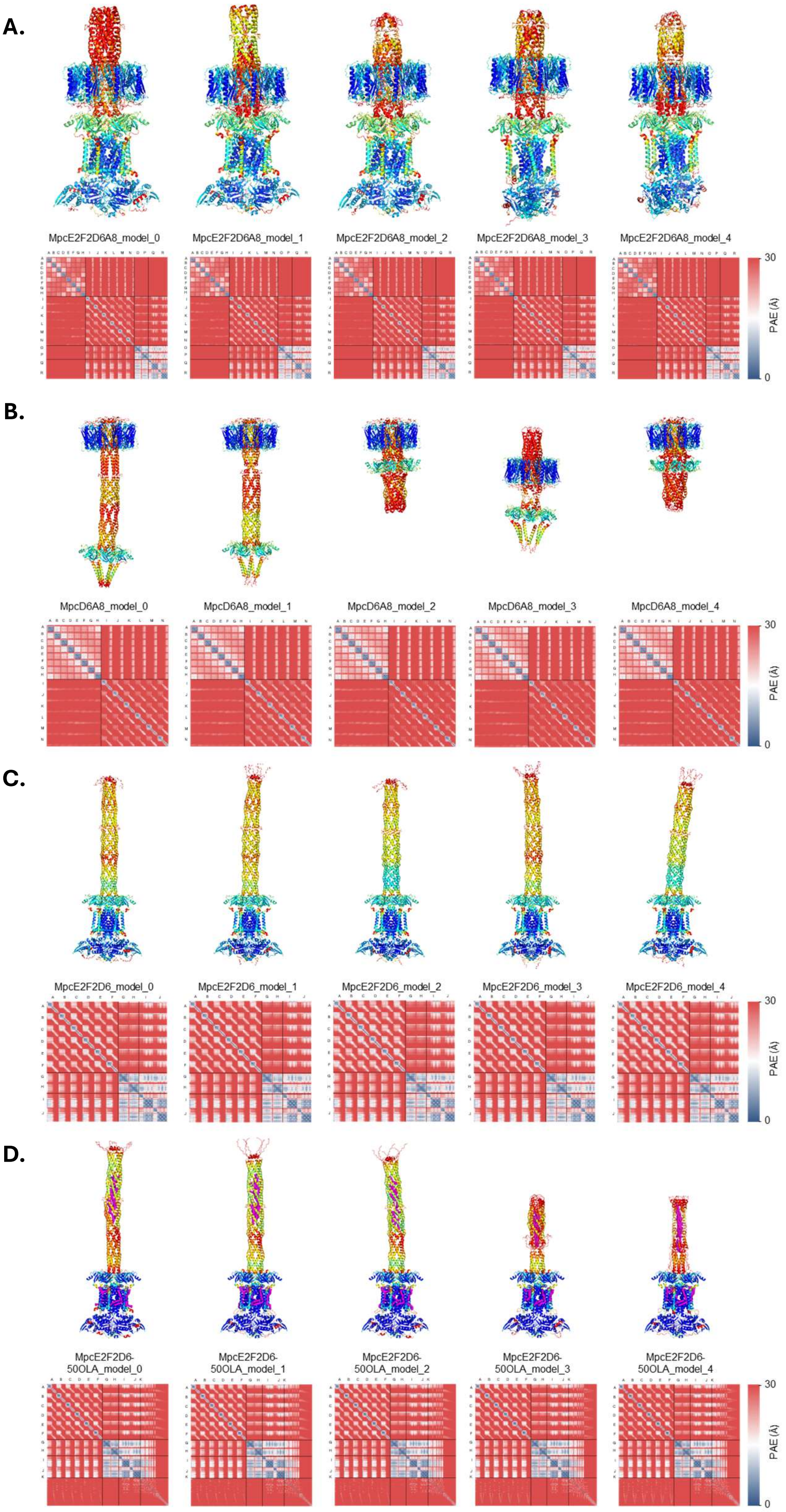

Appendix Figure S6. The 5 model interactions predicted for the Mpc complex. The AlphaFold3 multimer was applied to predicted molecular interactions. Predicted structures are coloured with the pLDDT confidence prediction (red pLDDT<50; yellow pLDDT>70; cyan 90>pLDDT>70; blue pLDDT>90).

At the bottom of each predicted structure complex are the associated Predicted Aligned Error (PAE) maps, generated on https://thecodingbiologist.com/tools/pae.html. The confidence in the relative positioning of the domains is indicated by the graphical representation of the PAE. Different forms of Mpc complex prediction were performed; **(A)** $MpcE_2F_2D_6A_8$ with the PAE regions corresponding to each chain of the MpcA octamer (from A to H, small letters along the square), of the MpcD hexamer (from I to N), of the MpcF dimer (O and P) and of the MpcE dimer (Q and R) are labelled. **(B)** The $MpcD_6A_8$ complex with the PAE regions corresponding to each chain of the MpcA octamer (from A to H) and of the MpcD hexamer (from I to N) are labelled. Finally, the **(C)** $MpcE_2F_2D_6$ with the PAE regions corresponding to each chain of the MpcD hexamer (from A to F), of the MpcF dimer (G and H) and of the MpcE dimer (I and J) are labelled, and **(D)** with 50 oleic acids (in pink) are labelled with the corresponding PAE regions (K). Given the limitations of the ligands proposed by AlphaFold3, the oleic acid, a fatty acid with an 18-carbon monounsaturated chain, was used to mimic the PLs. It is noteworthy that the predominant *B. abortus* PLs have 18-carbon chain FAs.

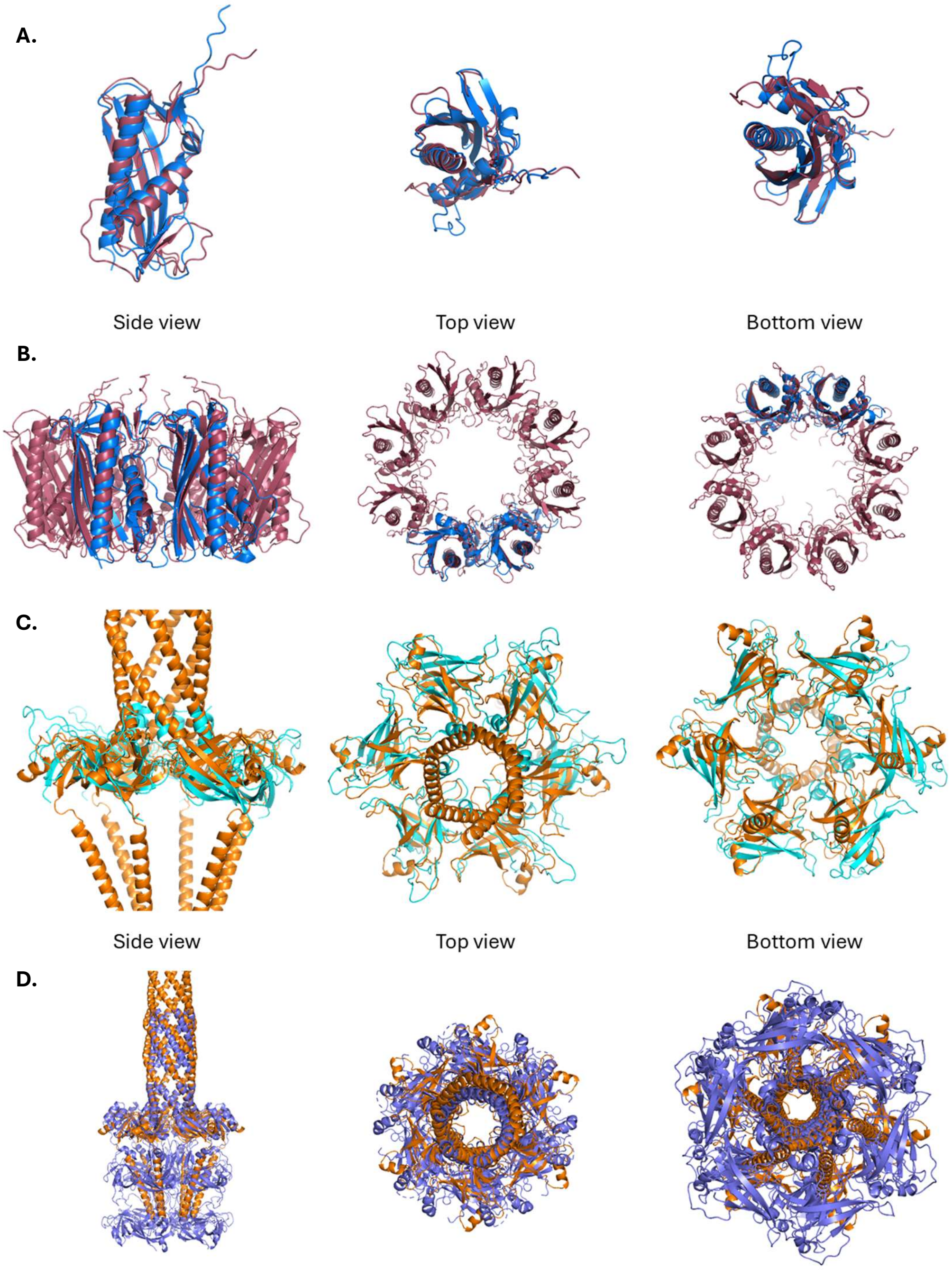

Appendix Figure S7. Structural comparison of MpcA/PqiC and MpcD/MlaD. A. Superimposition of predicted monomers by AlphaFold2 of PqiC from *E. coli* K12 (blue marine) and MpcA from *B. abortus* 544 (red raspberry). Superimposition (Super alignment function) was performed on PyMol, with a RMSD = 1.581. **B.** Alignment of dimeric structure of PqiC (X rays diffraction structure [8Q2C], blue marine) and the predicted structure of octameric form of MpcA by AlphaFold2 (red raspberry). Super alignment was performed on PyMol with a RMSD = 1.689. **C.** Alignment of hexameric structure of Mce domain of MlaD (electron microscopy [8OJG], cyan) and the predicted structure of hexameric form of MpcD by AlphaFold2 (orange). Super alignment was performed on PyMol with a RMSD = 1.947 **D.** Alignment of hexameric structure of PqiB (electron microscopy [5UVN], blue slate) and the predicted structure of hexameric form of MpcD by AlphaFold2 (orange). Super alignment of MCE domains (MpcD$_{S37\text{-}A138}$ and PqiB$_{H285\text{-}L431}$) was performed on PyMol with a RMSD = 0.923.
