# Supplementary figures and images for: A chimeric Mla-Pqi lipid transport system is required for Brucella abortus survival in macrophages
Source: EMBO J. 2025 Aug 13;44(18):5066–85. doi: 10.1038/s44318-025-00511-3 (PMC12436622; doi:10.1038/s44318-025-00511-3)

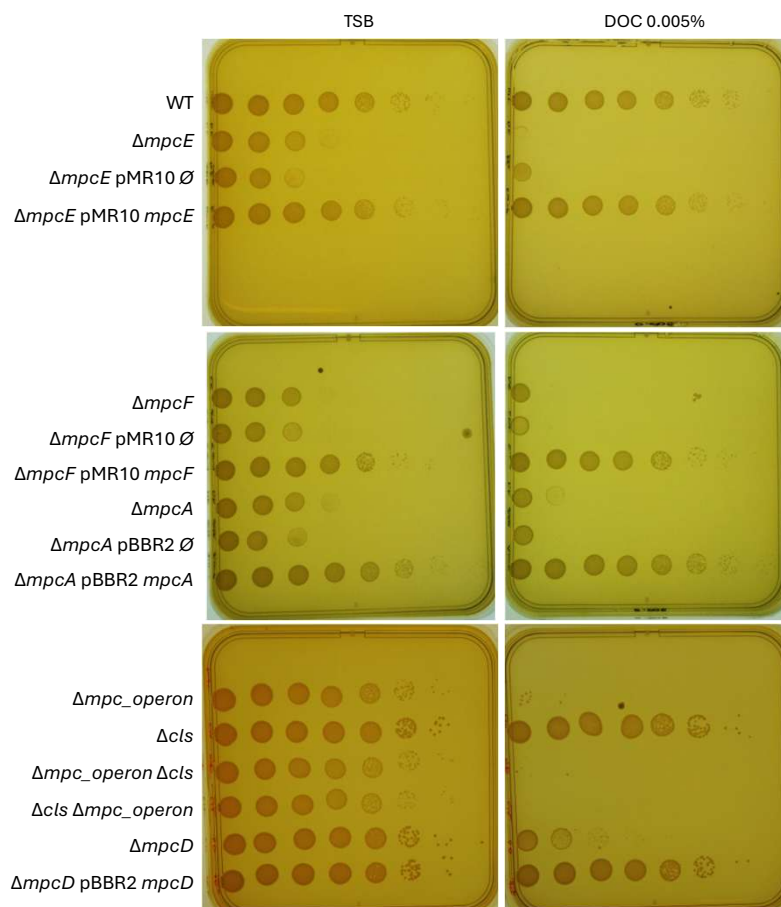

Supplement: Supplementary file 4 — Source data Fig. 2 [file 44318_2025_511_MOESM4_ESM.zip › SD_Figure2.pdf]

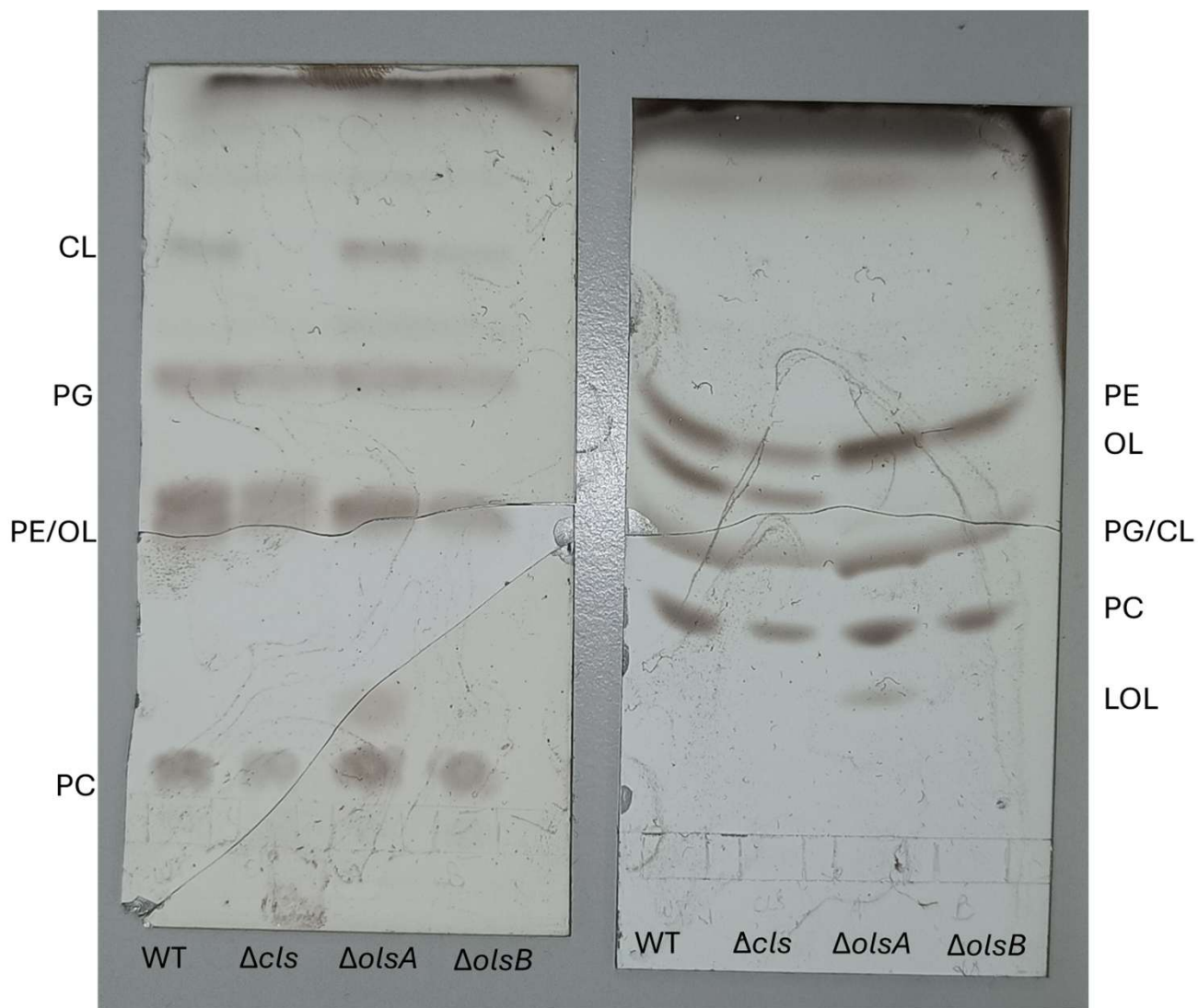

Supplement: Supplementary file 7 — Source data Fig. 5 [file 44318_2025_511_MOESM7_ESM.zip › SD_figure5/SD_Figure5A.pdf]

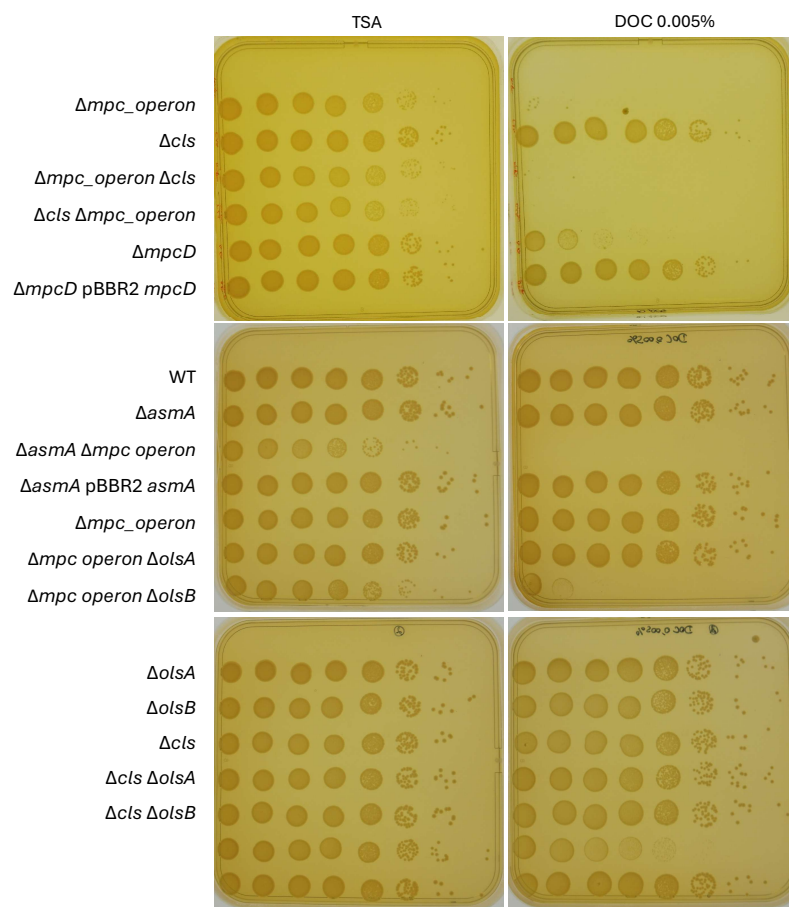

Supplement: Supplementary file 7 — Source data Fig. 5 [file 44318_2025_511_MOESM7_ESM.zip › SD_figure5/SD_Figure5B.pdf]

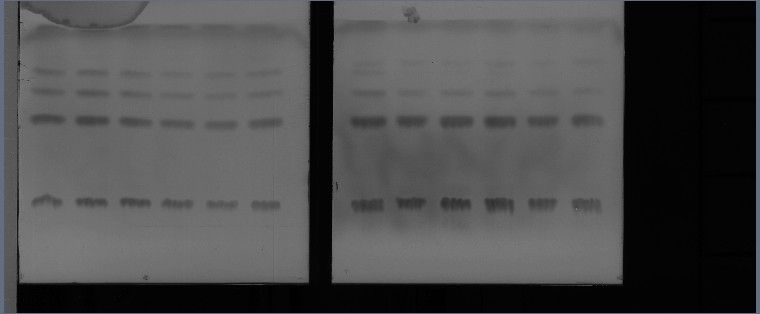

Supplement: Supplementary file 8 — EV and Appendix Figure Source Data [file 44318_2025_511_MOESM8_ESM.zip › SD_appendix-extanded_view/SD_Appendix-FigureS2.jpg]
