# Supplementary material for: A chimeric Mla-Pqi lipid transport system is required for Brucella abortus survival in macrophages
Source: EMBO J. 2025 Aug 13;44(18):5066–85. doi: 10.1038/s44318-025-00511-3 (PMC12436622; doi:10.1038/s44318-025-00511-3)
Supplement: Supplementary file 8 — EV and Appendix Figure Source Data [file 44318_2025_511_MOESM8_ESM.zip › SD_appendix-extanded_view/SD_FigureEV4A.pdf]

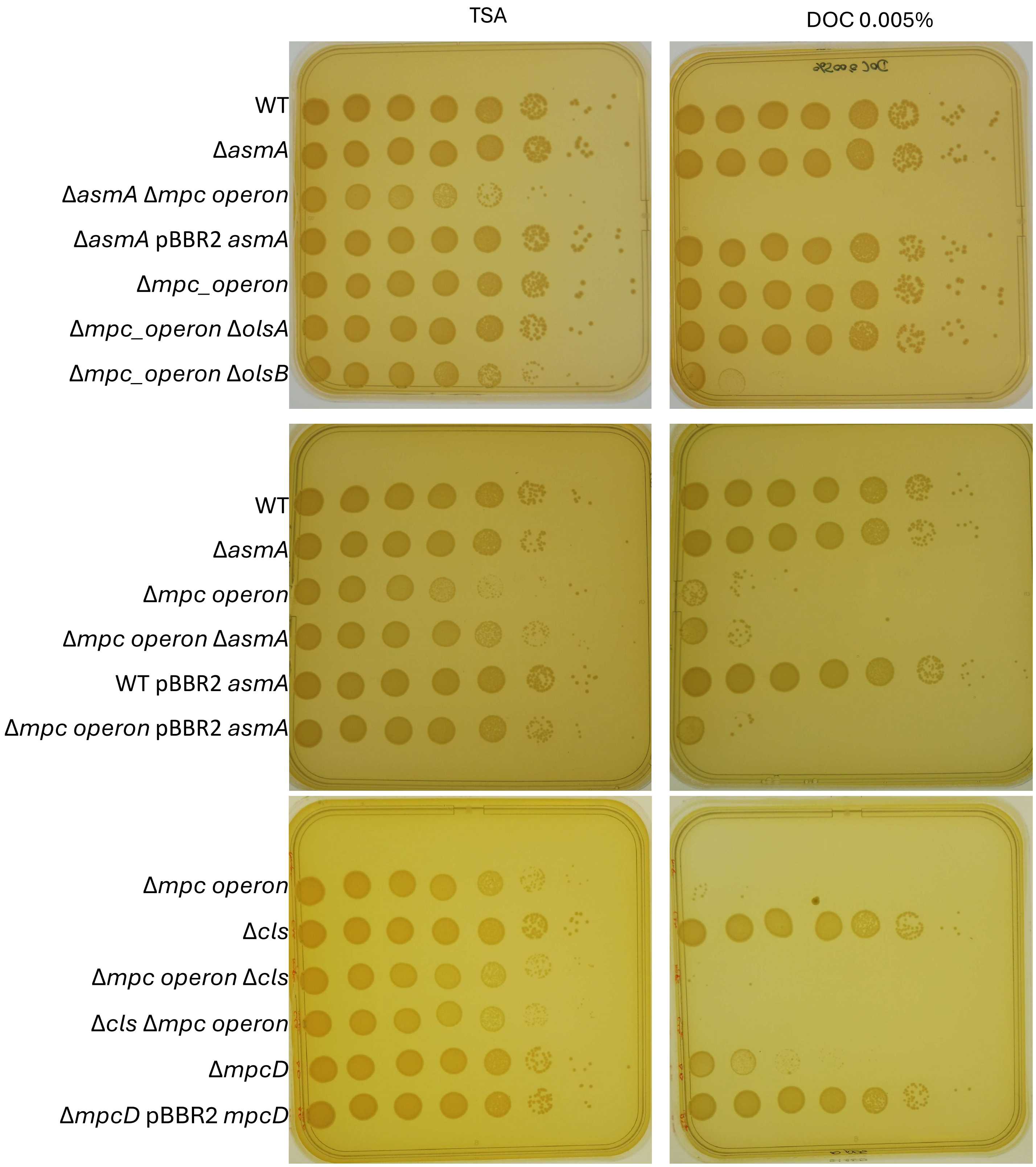
TSA
DOC 0.005%
WT
ΔasmA
ΔasmA Δmpc operon
ΔasmA pBBR2 asmA
Δmpc_operon
Δmpc_operon ΔolsA
Δmpc_operon ΔolsB
WT
ΔasmA
Δmpc operon
Δmpc operon ΔasmA
WT pBBR2 asmA
Δmpc operon pBBR2 asmA
Δmpc operon
Δcls
Δmpc operon Δcls
Δcls Δmpc operon
ΔmpcD
ΔmpcD pBBR2 mpcD
