# Supplementary material for: A chimeric Mla-Pqi lipid transport system is required for Brucella abortus survival in macrophages
Source: EMBO J. 2025 Aug 13;44(18):5066–85. doi: 10.1038/s44318-025-00511-3 (PMC12436622; doi:10.1038/s44318-025-00511-3)
Supplement: Supplementary file 9 — Expanded View Figures [file 44318_2025_511_MOESM9_ESM.pdf]

Expanded View Figures

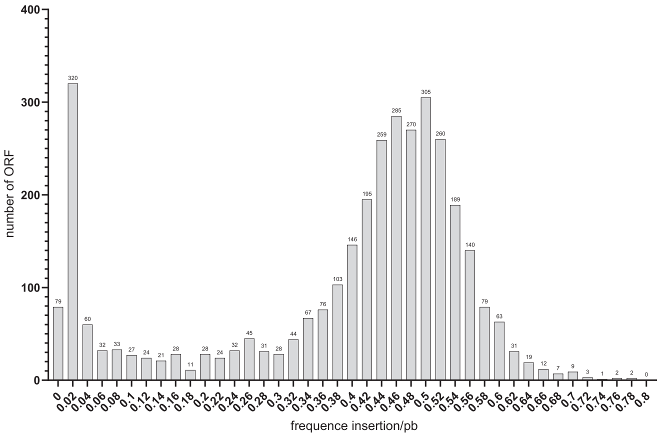

Figure EV1. Frequency distribution of the frequency of insertions per pair of bases for mini-Tn*5* across all open reading frames (ORFs) in the *B. abortus* genome.

For each mutated gene, a Transposon insertion frequency (TnIF) is computed. Transposon insertion sites were identified through Illumina sequencing ($n = 1$). The frequency of insertions per pb for each of 3390 ORFs from *B. abortus* genome are represented by classes of 0.02. Under 0.1 frequency of insertions per pb, the ORFs are considered essential for growth on TSA-rich medium, which corresponds to 16.25% of the predicted genes. Source data are available online for this figure.

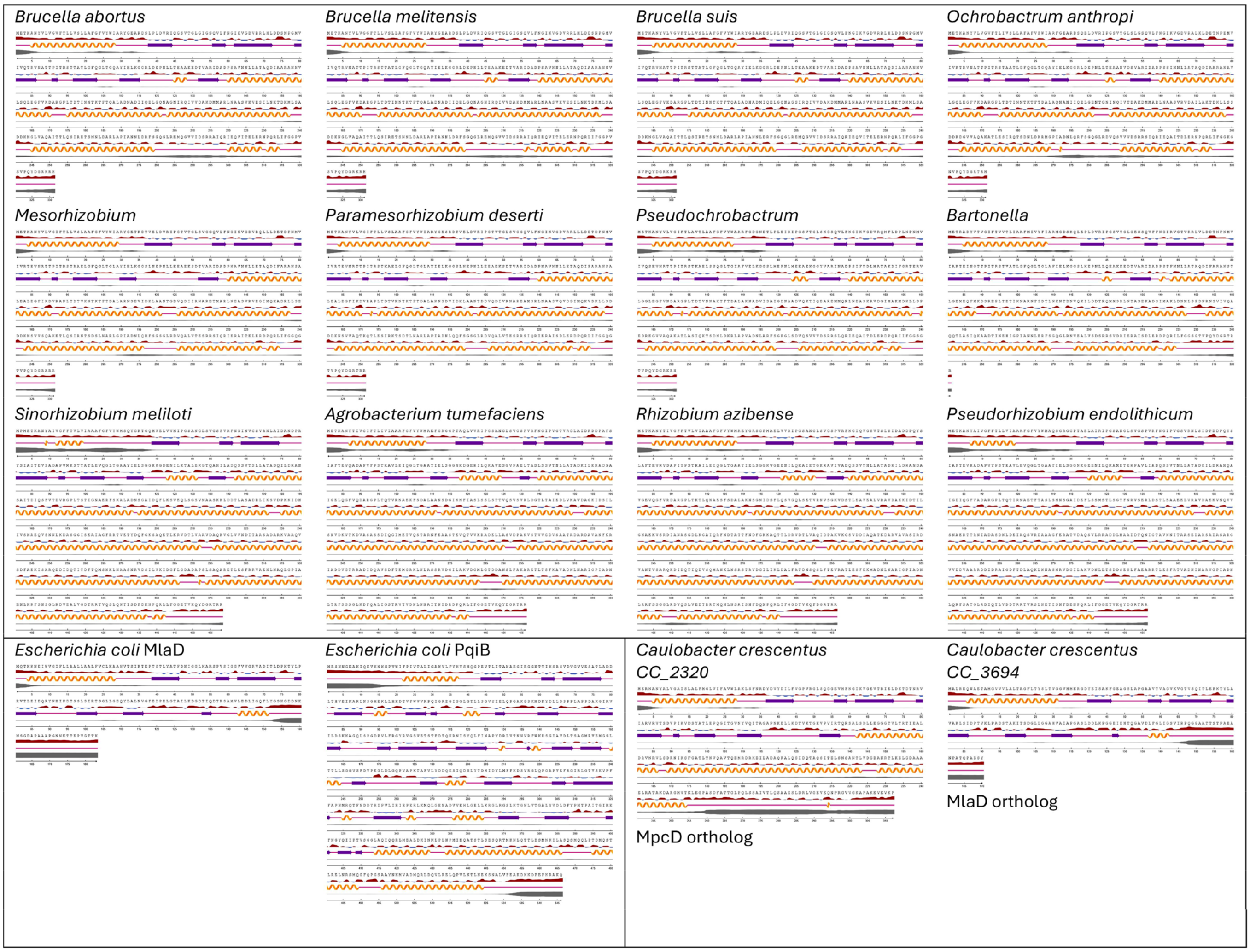

Figure EV2. Secondary structure prediction by NetSurfP-2.0 (Klausen et al, 2019) of MpcD and homologous proteins in some Hyphomicrobiales bacteria (top panel).

Secondary structure of MlaD and PqiB in *E. coli* (lower left panel) and secondary structure prediction of proteins in *Caulobacter crescentus* (lower right panel), one homologous to *B. abortus* MpcD and the other homologous to *E. coli* MlaD. Source data are available online for this figure.

 © The Author(s)

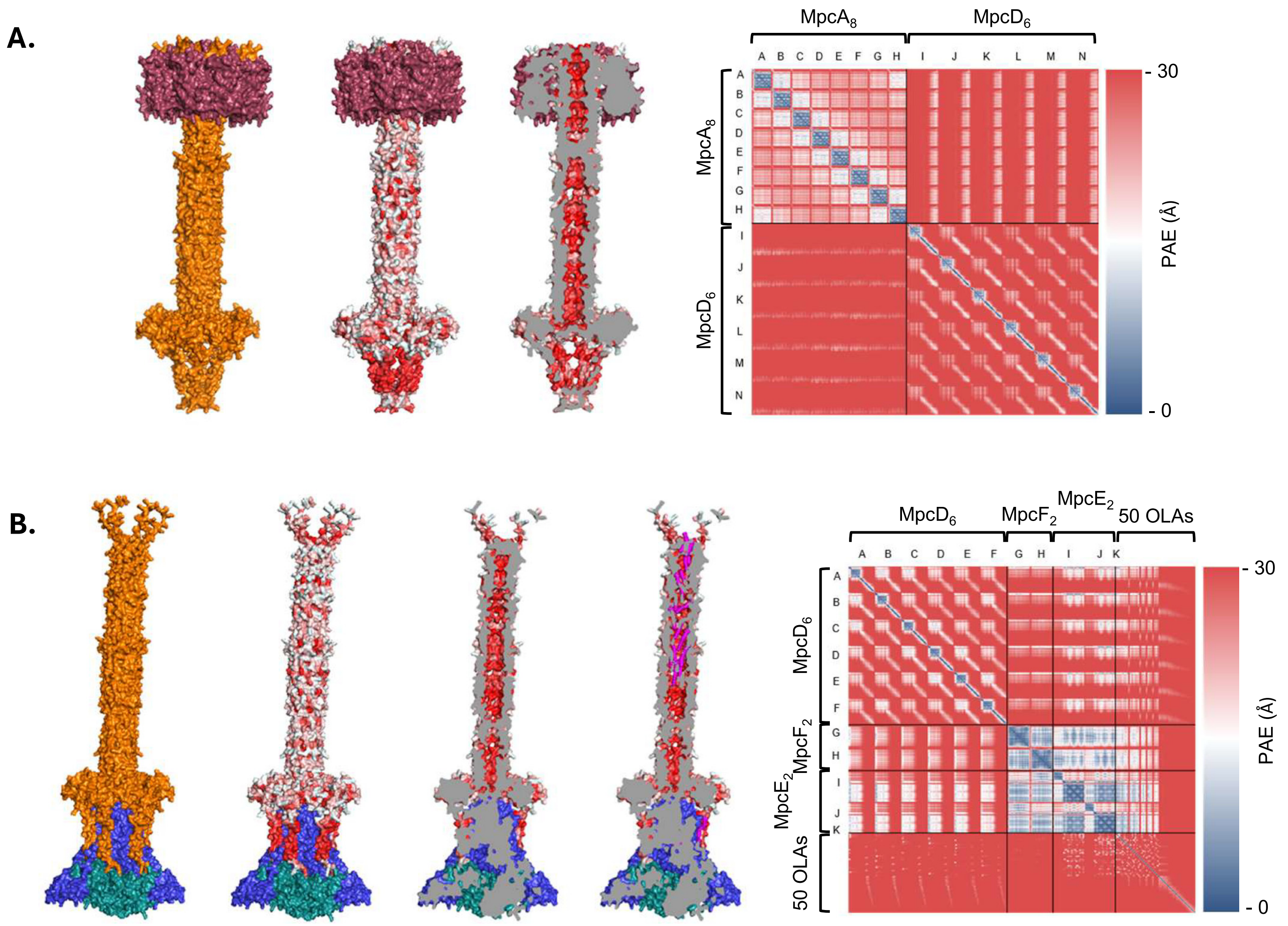

Figure EV3. The model predictions for $MpcD_6A_8$ (top) and $MpcE_2F_2D_6$ (bottom).

The surface representation predicted by the AlphaFold3 multimer of the biomolecular interactions of complexes of (**A**) $MpcD_6A_8$ (model_1) and (**B**) $MpcE_2F_2D_6$ (model_1) with the associated predicted aligned error (PAE) maps associated. The first panel shows the predicted interaction with the homodimer of MpcE (blue), the homodimer of MpcF (green), the homo-hexamer of MpcD (orange) and the homo-octamer of MpcA (pink). In the second panel, the MpcD homo-hexamer is coloured according to the Eisenberg hydrophobicity scale using the color_h command in PyMol (hydrophobic regions depicted in red). The third panel shows a slice of the complexes view, allowing observation of the predicted hydrophobicity of the full-length tunnel within the hexameric MpcD channel. The final panel shows the slice of the $MpcE_2F_2D_6$ complex in predicted interaction with 50 oleic acids (magenta), some of which are situated within the channel formed by the coiled-coil alpha-helices of MpcD.

© The Author(s)

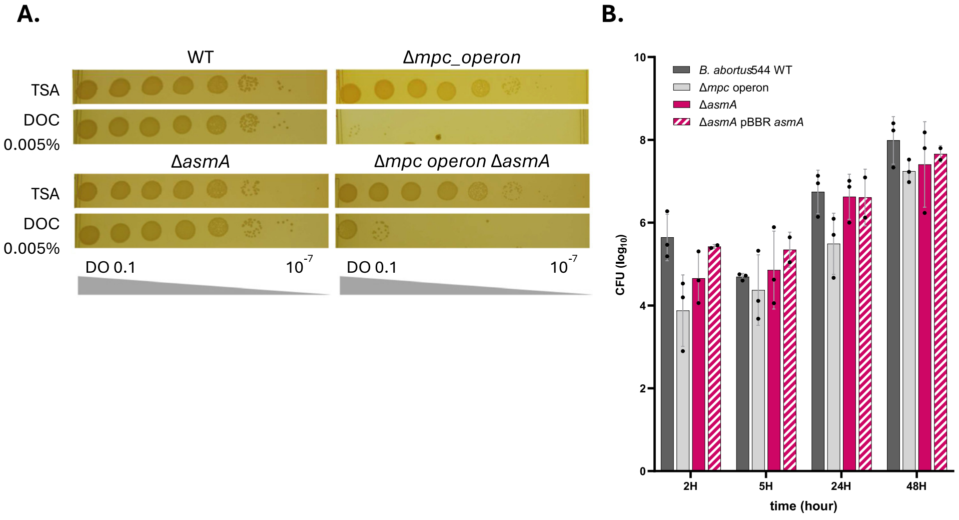

Figure EV4. The sensitivity phenotype of *asmA* deletion mutants.

(**A**) The plating assay on the *asmA* mutants, the *mpc* operon mutant and double mutants on DOC. All the mutants with deletion of *mpc* operon present the same sensitivity to DOC at 0.005%. The overnight cultures were normalised to 0.1 OD_{600} and serially diluted before being plated onto TSA with or without DOC. (**B**) Intracellular replication of WT, *mpc* operon, *asmA* mutant mutant and the double mutants were assessed by CFU at 2, 5, 24 and 48 h post-infection of J774.A1 macrophages. The data represents the mean ± SD and were compiled from three independent replicates, represented by the dots. Statistical significance between the results for a given strain and those for the WT was determined using a two-way ANOVA. No significant difference was found ($p \geq 0.05$). Source data are available online for this figure.

 © The Author(s)
